# Microbiota composition of the dorsal patch of reproductive male *Leptonycteris yerbabuenae*

**DOI:** 10.1371/journal.pone.0226239

**Published:** 2019-12-16

**Authors:** Osiris Gaona, Daniel Cerqueda-García, Luisa I. Falcón, Guillermo Vázquez-Domínguez, Patricia M. Valdespino-Castillo, Carla-Ximena Neri-Barrios

**Affiliations:** 1 Posgrado en Ciencias Biológicas de la Universidad Nacional Autonóma de México, Instituto de Ecología, UNAM, Mexico City, México; 2 Laboratorio de Ecología Bacteriana, Instituto de Ecología, UNAM, Parque Científico y Tecnológico de Yucatán, Mérida, Yucatán, México; 3 Consorcio de Investigación del Golfo de México (CIGOM), Centro de Investigación y de Estudios Avanzados del Instituto Politécnico Nacional, Unidad Mérida, Departamento de Recursos del Mar, Mérida, Yucatán, México; 4 Laboratorio de Ecología Funcional, Instituto de Investigaciones en Ecosistemas y Sustentabilidad, Universidad Nacional Autónoma de México, Morelia, Michoacán, México; 5 Climate and Ecosystem Sciences Division, Lawrence Berkeley National Laboratory, University of California, Berkeley, California, United States of America; University of Illinois at Urbana-Champaign, UNITED STATES

## Abstract

Bacteria and other types of microbes interact with their hosts in several ways, including metabolic pathways, development, and complex behavioral processes such as mate recognition. During the mating season, adult males of the lesser long-nosed agave pollinator bat *Leptonycteris yerbabuenae* (Phyllostomidae: Glossophaginae) develop a structure called the dorsal patch, which is located in the interscapular region and may play a role in kin recognition and mate selection. Using high-throughput sequencing of the V4 region of the 16S rRNA gene, we identified a total of 2,847 microbial phylotypes in the dorsal patches of eleven specimens. Twenty-six phylotypes were shared among all the patches, accounting for 30 to 75% of their relative abundance. These shared bacteria are distributed among 13 families, 10 orders, 6 classes and 3 phyla. Two of these common bacterial components of the dorsal patch are *Lactococcus* and *Streptococcus*. Some of them—*Helcococcus*, *Aggregatibacter*, *Enterococcus*, and Corynebacteriaceae—include bacteria with pathogenic potential. Half of the shared phylotypes belong to *Gallicola*, *Anaerococcus*, *Peptoniphilus*, *Proteus*, *Staphylococcus*, *Clostridium*, and *Peptostreptococcus* and specialize in fatty acid production through fermentative processes. This work lays the basis for future symbiotic microbe studies focused on communication and reproduction strategies in wildlife.

## Introduction

The microbiome is the assemblage of archaea, bacteria, viruses and other microorganisms associated with any multicellular organism (the host) in a particular environment [1,2]. The microbiome is a vital component in the evolution of the host, and in vertebrates it plays essential roles in almost all of the organism’s functions [3–7]. The relationships between the animal host and its microbiome are, for the most part, mutually beneficial [8]. Gut and skin microbiota have coevolved with their hosts, and this mutualism has led to the evolution of intra- and interspecific interactions [9,10]. Microbiota associated with skin and glands in mammals are and diverse, and host species is an important predictor of the microbial community assemblage [11]. It has been demonstrated that microbial communities play a role in the production of odors and chemical signals, which are important in species recognition, kin recognition, and mate choice [12–15]. The interaction between host and microbiome can thus alter the host’s social behavior at various points during its life cycle [14,15].

The role of bacteria in the processes of fermentation and production of volatile fatty acids has been previously reported in studies of canids and bats [16,17]. These short chain fatty acids, produced by bacteria, are the precursors of chemical signals, known as scents, which influence the intraspecific interactions of many mammals. Bacteria associated with the skin contribute to the host’s scent through the direct production of odors and the fermentation of organic compounds produced endogenously by the host, which is known as the “fermentative hypothesis” [18,19]. The fermentative hypothesis is based on two main assumptions: first, that volatile odorants are produced by bacteria that colonize mammal scent glands and ephitelial tissues and second, that individual “odor prints” are generated by differences among individuals in the composition of their bacterial communities [20].

Scent production as a consequence of bacterial fermentation has been reported in several bat species [17,21,22], most often among reproductive males [22–24]. Some examples of bats that possess odor-producing structures colonized by bacteria are the piscivorous *Noctilio leporinus* (Noctilionidae), which have a typical strong odor associated with *Staphylococcus aureus* [17], male sac-winged bats, *Saccopteryx bilineata* (Emballonuridae), which have a scent pouch that contains an odoriferous liquid used in courtship [17,25], and the big brown bat *Eptesicus fuscus* (Vespertilionidae), in which roost-mates have a shared odor signature [21]. Adult males of the nectar-feeding bats *Leptonycteris curasoae* and *Leptonycteris yerbabuenae* develop an oderiferous “dorsal patch”, a temporary structure in the interscapular region that contains fatty acids and signals mating readiness [22,26,27].

**Fig 1:**
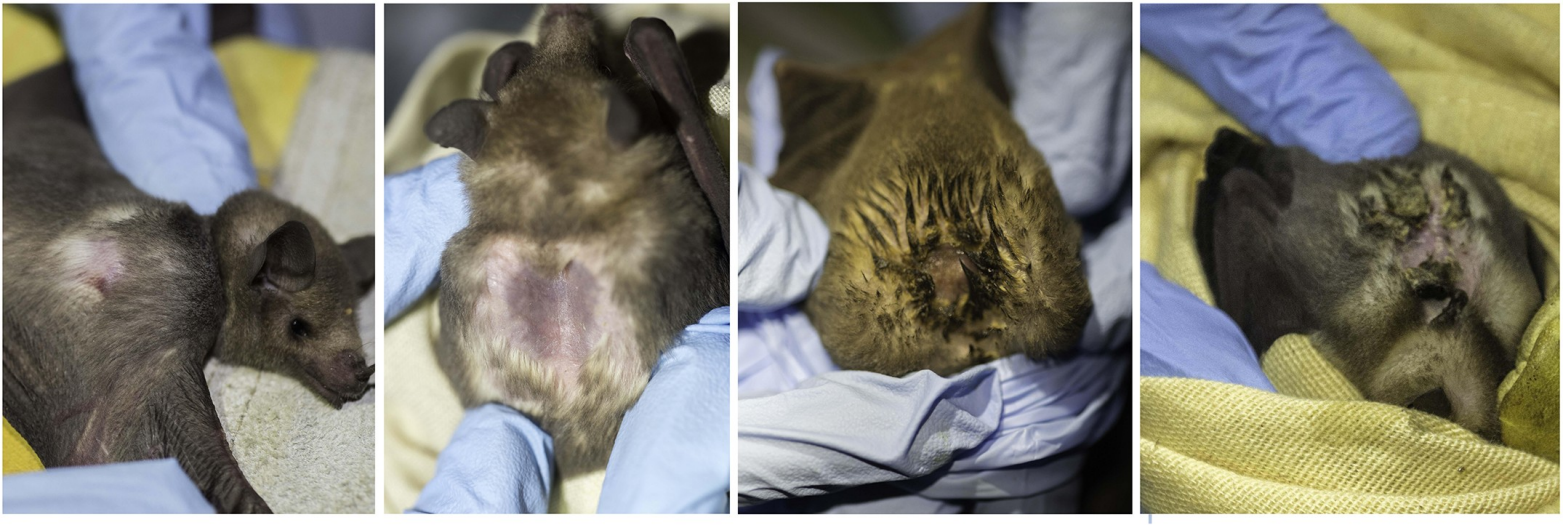
Development of dorsal patch in *L*. *yerbabuenae*. The dorsal areas of different male bats captured during the same sampling period show patches at different stages of development from a less developed patch (left) to a mature, well-developed patch (right).

Male *L*. *yerbabuenae* develop the odoriferous dorsal patch for a short time during the breeding season, using their forelimbs to spread fluids from the anus, penis, and mouth onto their backs in a behavior known as “smearing” [22,28] (Fig 1). The fact that all males with dorsal patches have enlarged testes, and that males with small testes do not develop this trait, suggests a strong association between dorsal patch development and sexual maturity and possibly readiness for reproduction [22–24,27]. This dorsal patch has a strong odor and a complex chemical profile [22,27], made of compounds that have been reported to be important during the reproductive season of *L*.*yerbabuenae* and *Leptonycteris curasoae* in female attraction [28,29]. Thus, overall, the available evidence suggests that the dorsal patch is involved in female mate choice mediated through odor [22,24,26,27]. Males with dorsal patches had fewer ectoparasites than those without them, suggesting that this structure might function as a dual signal (odoriferous and visual) of mating readiness and health status [22,26–28]. It has been hypothesized that the odor of the dorsal patch is an honest signal of health status [26].

In this study, we aimed to evaluate the microbial diversity associated with the dorsal patch of reproductive *L*. *yerbabuenae* males using a high throughput 16S rRNA amplicon sequencing approach.

## Materials and methods

### Study site

**Fig 2:**
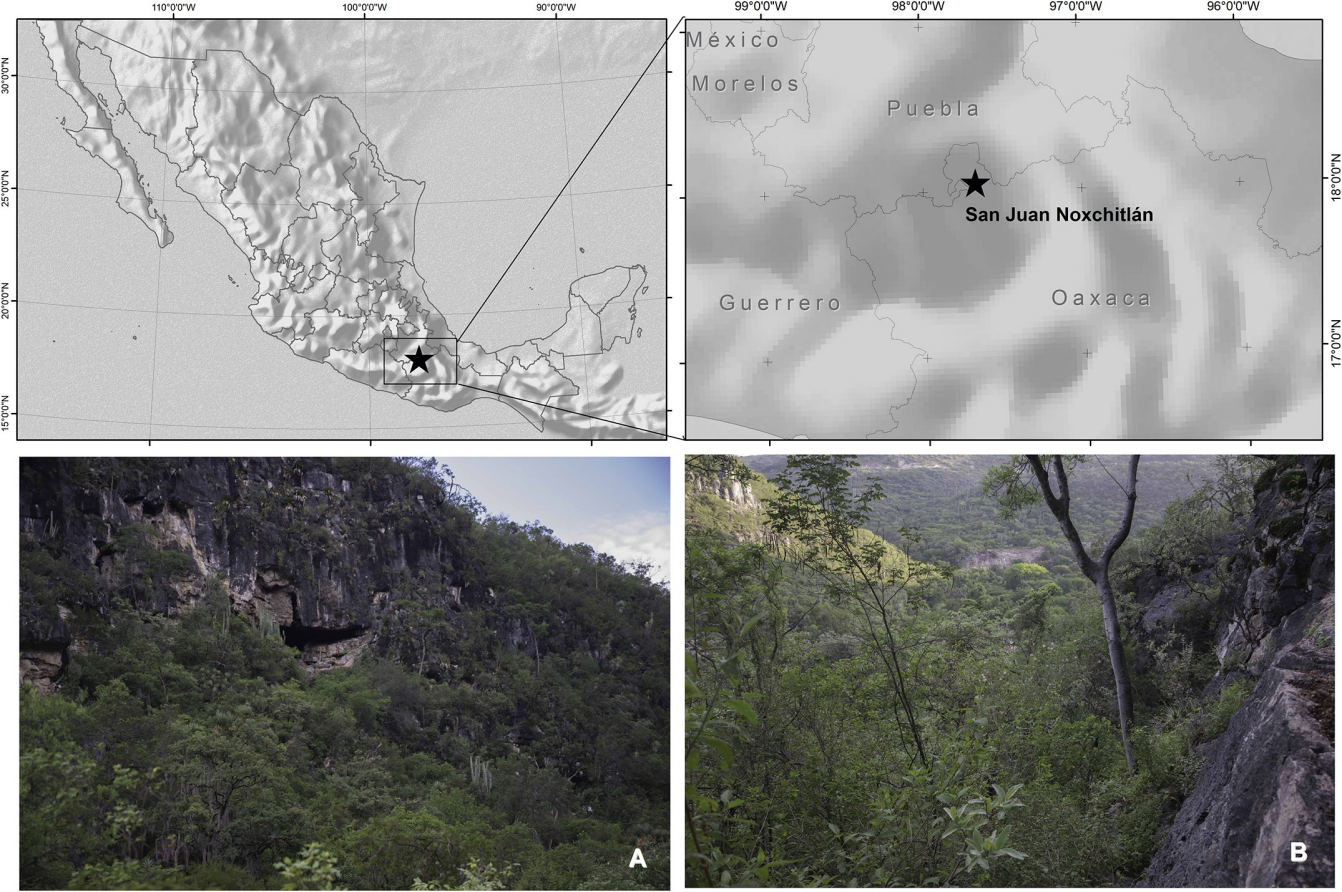
Location of San Juan Noxchitlan cave, Oaxaca, Mexico (18° 03’ 00.0” W and 97° 40’ 00.0” N), at an approximate altitude of 1978 m.a.s.l.

Bats were sampled in the San Juan Noxchitlan cave, Oaxaca (18° 03’ 00.0” W and 97° 40’ 00.0” N), at an altitude of 1978 m.a.s.l. [30–32] (Fig 2). The resident colony is comprised of approximately 100,000 bats [31]. This bachelor cave is located in the Tehuacan Valley, characterized as an isolated, arid-semiarid region (10,000 km^2^). Average annual rainfall is 495 mm, and annual mean temperature is 21 °C, with very rare frosts [33]. The vegetation is mainly tropical deciduous forest [34].

### Bat sampling

This study was conducted during the mating season in June 2015. Bats were captured using the methods described by Kunz et al. [35], using 12 m mist nets (Avinet, Dryden, New York, USA) placed at the entrance of the cave from 18:00h in the evening to 06:00h the next day, a sampling effort of 12 hours.

Interscapular dorsal patch samples (n = 11, named with the prefix Oax) were obtained following the guidelines of the American Society of Mammalogists for capture, handling and care of mammals [36,37]. To minimize animal suffering and distress, each animal was processed by experts only, ensuring effective and harmless handling. No anesthetics were administered for chemical immobilization, as they were deemed unnecessary and would only increase the mortality risk. Samples of the crust and fur of the interscapular patch were taken using gloves and sterile surgical calipers and scissors. A 0.25 cm^2^ area of the formed crust and hair was cut without touching the skin, and placed into sterile Eppendorf tubes (1.5 ml). Samples were frozen until processing using a field liquid nitrogen dewar.

### Ethics statement

Samples were taken from wild bats that were released in the same area as capture, causing no apparent harm to individuals. *Leptonycteris yerbabuenae* is not under federal protection by Mexican law (NOM-059-SEMARNAT-2010). Scientific collection activities were carried out under a scientific collection permit number granted by the Mexican Subsecretary of the Environment and Natural Resources (SEMARNAT), number FAUT-0231, SGPA/DGVS/05780/15. SEMARNAT specifically approved and authorized the tissue sampling methods under this collection permit. Laboratory activities were carried out in the Ecology Institute of the Universidad Nacional Autonoma de Mexico (UNAM), under the authorization of the Biosafety Commission of the Ecology Institute, UNAM; no specific permit was needed because only tissue and skin samples were used (no *in vivo* studies were included). All biosafety standard requirements from the Ecology Institute were satisfied.

### DNA extraction

DNA was extracted using the DNeasy Blood & Tissue kit (Qiagen, Valencia, CA) with some modifications. Dorsal patch samples were diluted with 180 μl of Animal Tissue Lysis (ATL) extraction buffer and incubated with lysozyme A (30 mg/ml) and proteinase K (10 mg/ml). After enzymatic digestion, the manufacturer’s protocol was followed. DNA was precipitated with 1 volume of chilled absolute ethanol and 0.1 volume of 3 M sodium acetate, then washed with 70% ethanol. Finally, DNA was eluted in 30 μl of molecular grade water and stored at -20°C prior to PCR amplification.

### 16S rRNA gene amplification and sequencing

DNA samples were PCR amplified with universal bacteria/archaeal primers 515F/806R (hypervariable region V4) following the procedures reported by Caporaso et al. [38]. PCR reactions (25 μl) contained 2–6 ng of total DNA, 2.5 μl Takara ExTaq PCR buffer 10X, 2 μl Takara dNTP mix (2.5 mM), 0.7 μl bovine serum albumin (BSA, 20 mg ml^-1^), 1 μl primers (10 μM), 0.125 μl Takara Ex Taq DNA Polymerase (5 U μl^-1^; TaKaRa, Shiga, Japan) and nuclease-free water. Samples were amplified in triplicate using a PCR protocol that included an initial denaturation step at 95°C (3 min), followed by 35 cycles of 95°C (30 s), 52°C (40 s) and 72°C (90 s), followed by a final extension 72°C (12 min). Triplicates were then pooled and purified using the SPRI magnetic bead, AgencourtAMPure XP PCR purification system (Beckman Coulter, Brea, CA, USA). The purified 16S rRNA fragments (~20 ng per sample) were sequenced on an Illumina MiSeq platform (Yale Center for Genome Analysis, CT, USA), generating ~250 bp paired end reads. The sequence data are available from the NCBI Bioproject PRJNA496019; SUB4625080.

### Sequence analysis

The 2x300 MiSeq Illumina paired-end reads were overlapped and merged using FLASH [38,39]. Nucleotide sequences were processed in the QIIME pipeline [40]. Quality filtering and demultiplexing were done as suggested by Caporaso et al. [40] and Bokulich et al. [41] (Q = 19, p = 0.75, r = 3, n = 0). Sequences were then clustered into Operational Taxonomic Units (OTUs) at 97% sequence identity in the open reference mode with USEARCH 6.1[42]. Chimeras were removed using UCHIME2 [43] and OTUs were taxonomically assigned with UCLUST, using the Greengenes database (release 13_5_8)[44]. Sequences were rarefied to 19 000. The taxonomic abundance and statistical analyses were plotted in R with the phyloseq [45] and ggplot2 [46] packages.

OTUs that were shared among samples were searched for their closest OTU using a BLAST search against the RefSeq-NCBI database. The three best hits for each OTU were aligned with MUSCLE [47], and a phylogenetic tree was built with PHYML software [48], using the GTR substitution model and 1000 bootstraps.

## Results

A total of 17 male *L*. *yerbabuenae* individuals were captured, but only 11 samples met the minimal requirements for high-quality DNA extraction. Patches were found on mature males only and covered between 2 and 4 cm^2^ of the interscapular area.

### Microbiota diversity and composition

**Fig 3:**
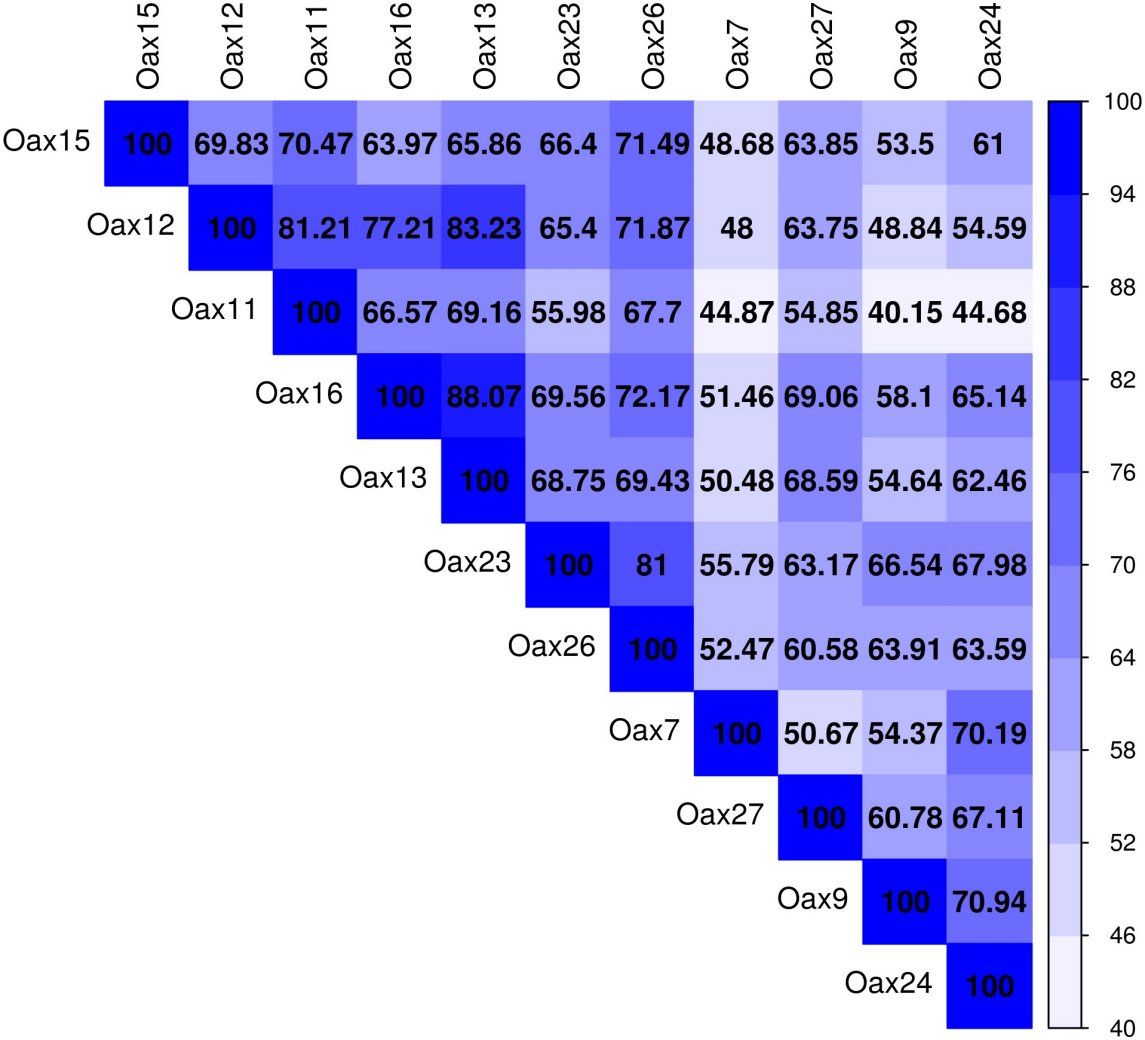
Graphical representation of similarity among the samples from each of the 11 individuals base on weighted unifrac distance.

**Fig 4:**
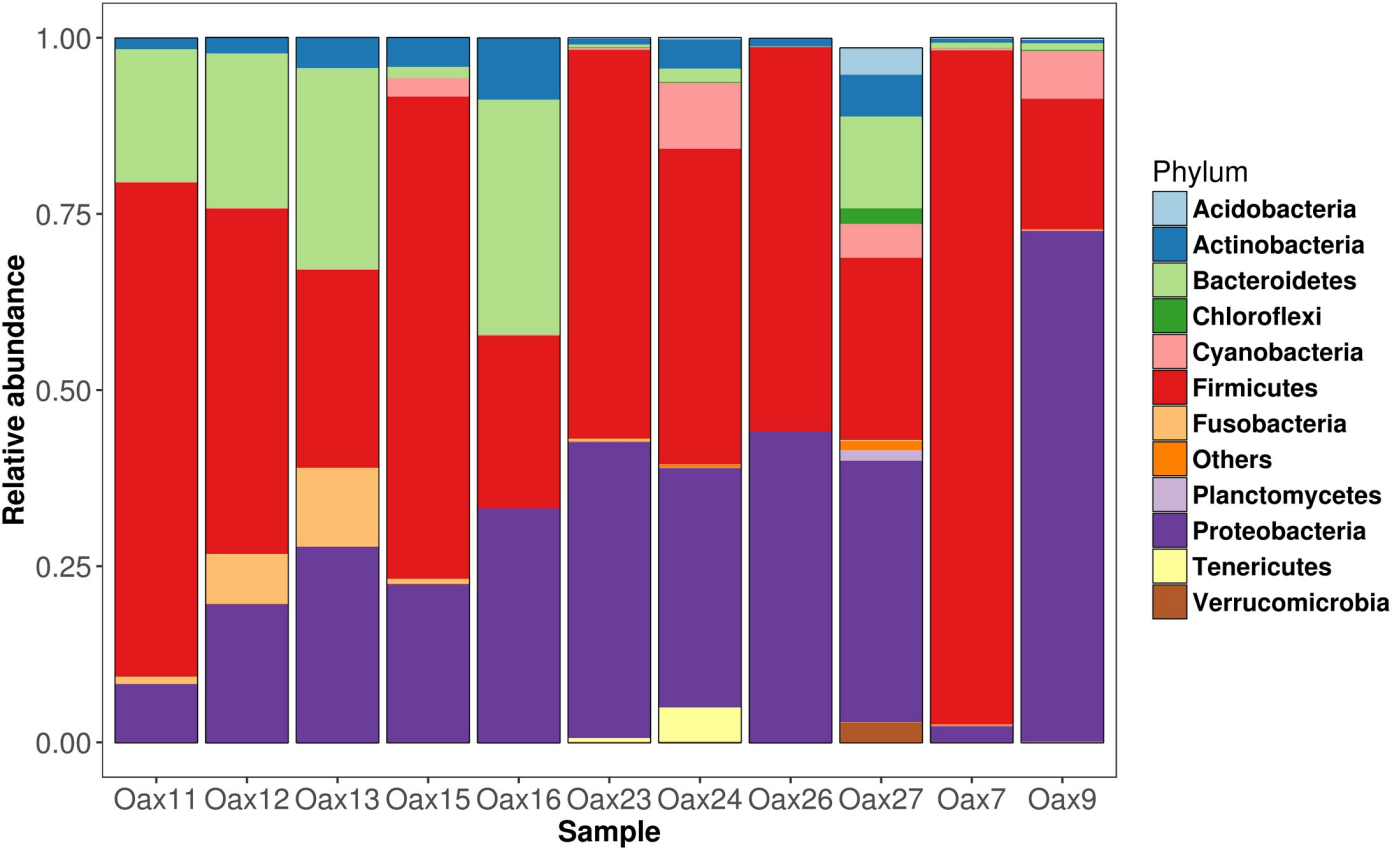
Composition of the dorsal patch at the phylum level.

**Fig 5:**
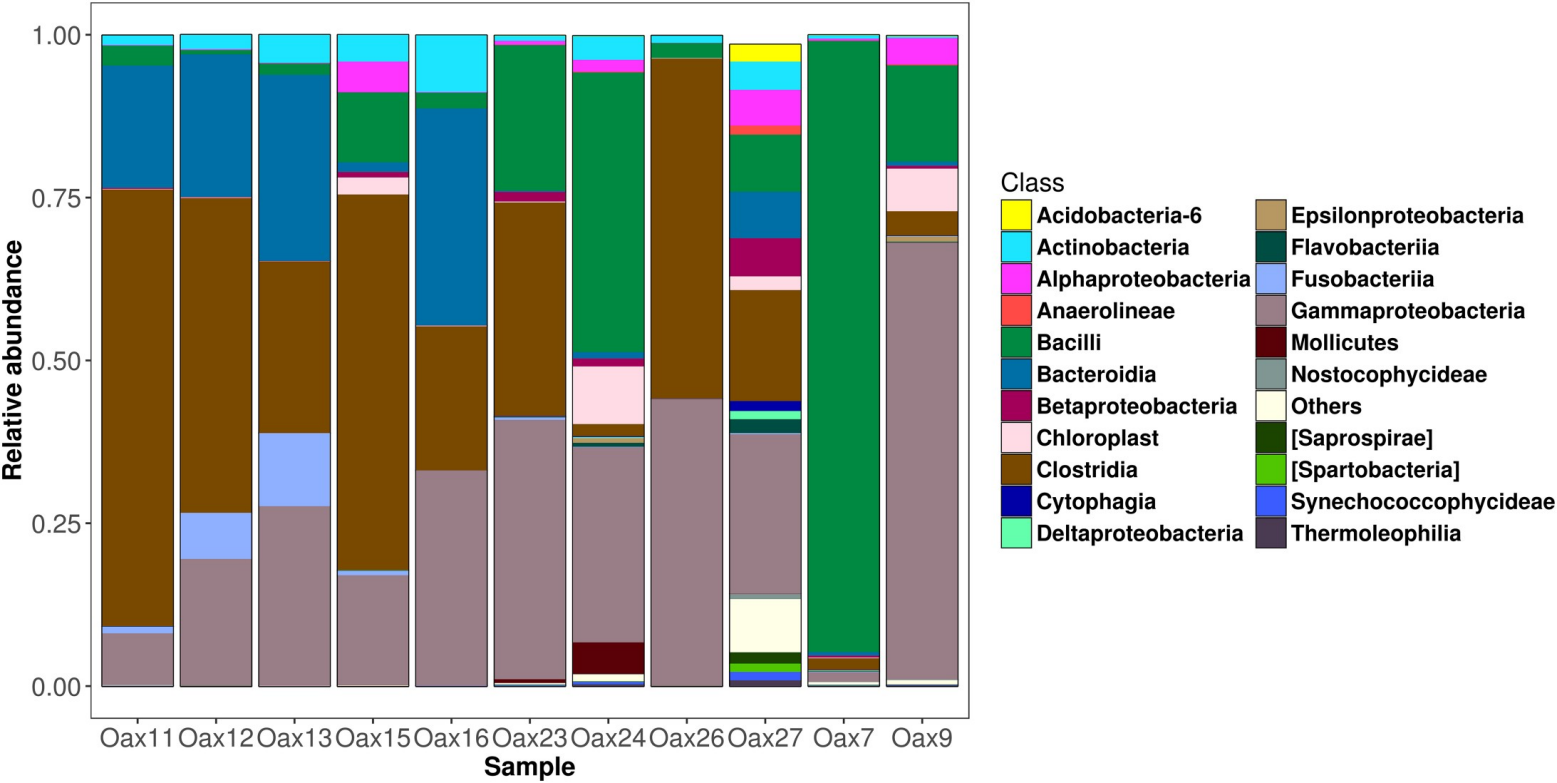
Composition of the dorsal patch at the class level.

**Fig 6:**
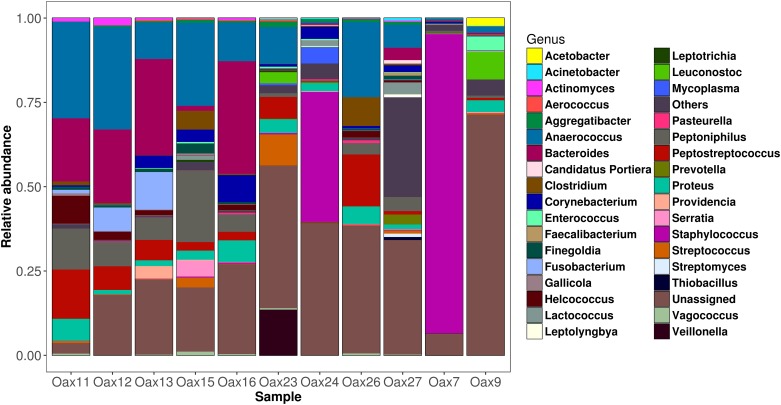
Composition of the dorsal patch at the genus level.

In total for the eleven sampled dorsal patches, we found 2,847 phylotypes. The number of observed phylotypes and diversity indices (Shannon and Chao 1) for each individual are provided in Table 1, and similarity among samples based on weighted unifrac distances are given in Fig 3. While there were no substantial differences in bacterial groups, the abundance and composition of bacteria varied among individuals (Figs 4–6).

**Table 1:** Alpha diversity of phylotypes in the microbiota from each of the 11 dorsal patch samples.

SAMPLE	SHANNON INDEX	Observed OTUs	CHAO1
**OAX9**	4.55	552	932.68
**OAX23**	5.00	639	1201.76
**OAX26**	4.23	523	1245.50
**OAX24**	4.39	516	857.00
**OAX12**	4.51	521	1113.88
**OAX7**	2.02	415	566.08
**OAX15**	5.39	641	1287.01
**OAX11**	4.55	464	699.01
**OAX16**	4.48	529	1017.95
**OAX13**	4.56	437	909.35
**OAX27**	7.67	715	946.03

The dorsal patch microbiota of *L*. *yerbabuenae* was mainly composed of Firmicutes (48%) and Proteobacteria (36%), with smaller contributions from Actinobacteria (3.6%), Fusobacteria (2.8%), Cyanobacteria (2.4%), Tenericutes (0.5%), Bacteroidetes (0.4%), Verrumicrobia (0.03%), and 6.2% corresponding to unassigned bacteria (Fig 3). Class composition was highly skewed towards Gammaproteobacteria (40.44%), Clostridia (33.70%) and Bacilli (13.72) with minor contributions from Fusobacteria (2.06%), Alphaproteobacteria (1.84%), Betaproteobacteria (0.76%), Mollicutes (0.49%) and Actinobacteria (0.06%) (Fig 5).

The OTU level classification of the dorsal patch in *L*. *yerbabuenae* resulted in 102 recognized OTUs, but over 80% of OTUs were unassigned. The 20 most abundant identified species were: *Acinetobacter johnsonii*, *Actinomyces europaeus*, *Aggregatibacter segnis*, *Brevinema andersonii*, *Bulleidia p-1630-c5*, *Candidatus Nitrososphaera*, *Clostridium perfringens*, *Corynebacterium variabile*, *Faecalibacterium prausnitzii*, *Haemophilus parainfluenzae*, *Lactococcus garvieae*, *Leptolyngbya frigida*, *Methylobacterium adhaesivum*, *Morganella morganii*, *Pasteurella multocida*, *Serratia marcescens*, *Staphylococcus epidermidis*, *Staphyloccocus sciuri*, and *Veillonella parvula* (Fig 6).

**Fig 7:**
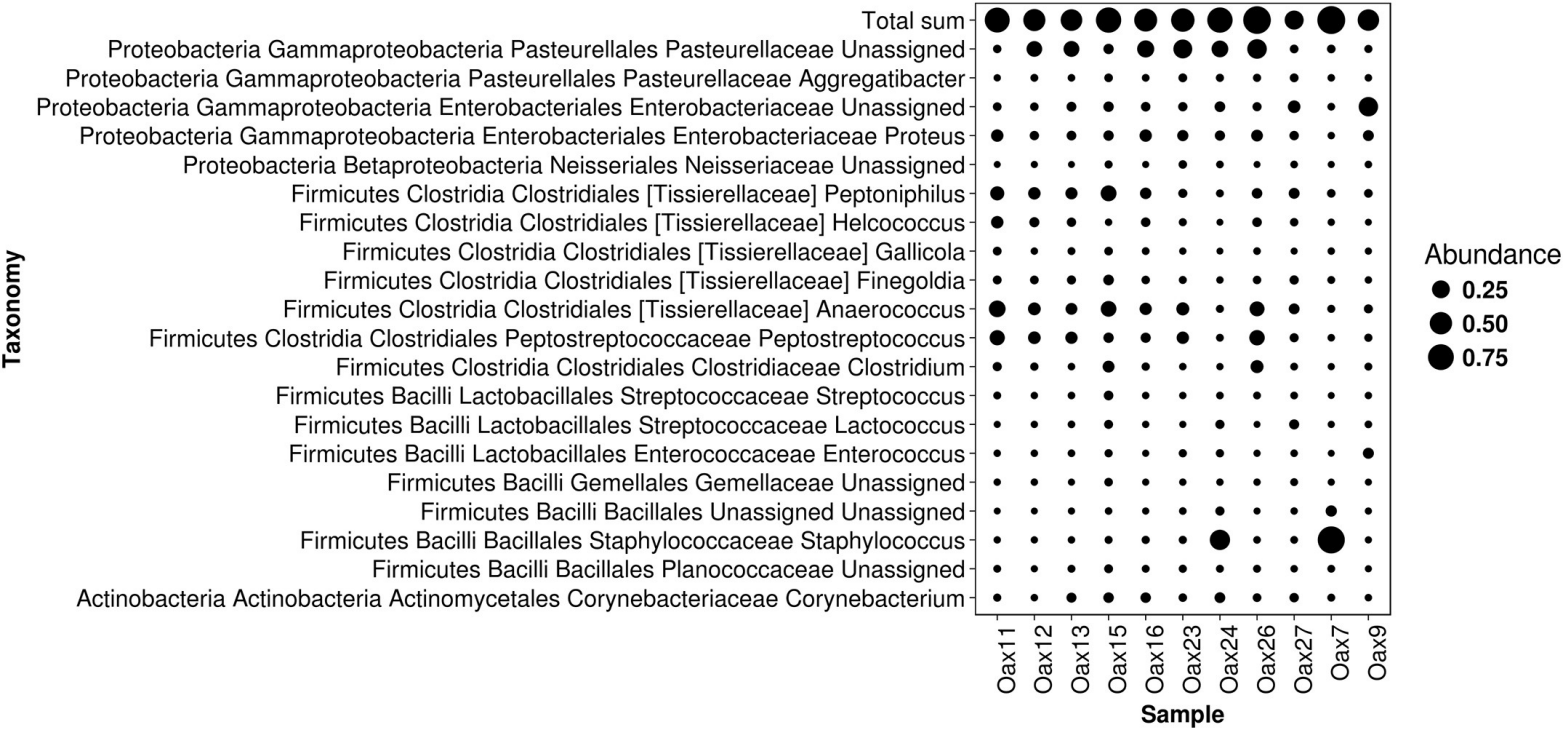
OTUs that were shared among all the samples. Dot sizes represent their relative abundance within the samples. These 26 OTUs combined contribute between 30 and 75% of the total abundance.

**Fig 8:**
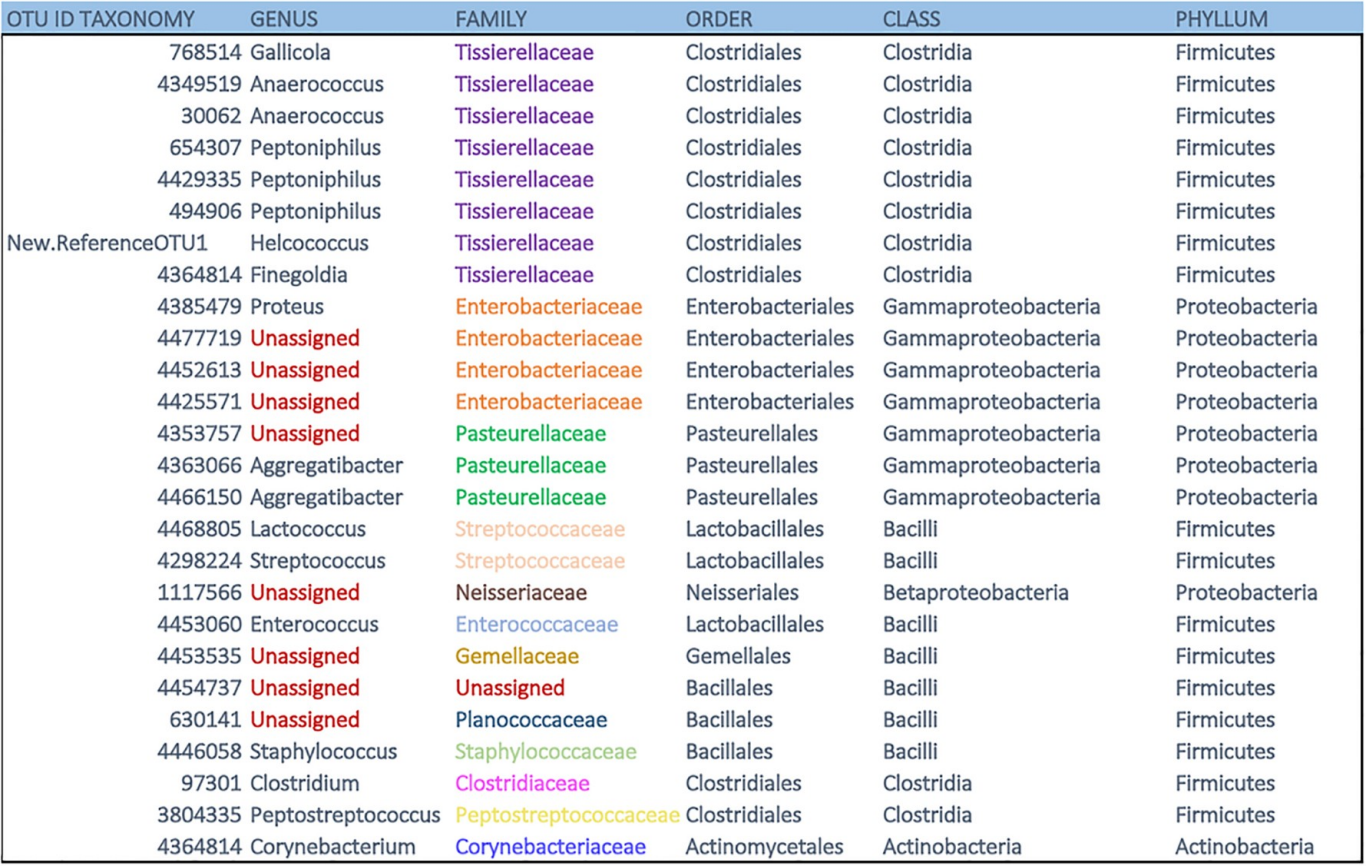
OTU ID taxonomy. Eight (31%) of the genera and one family were unassigned out of the 26 shared OTUs found in the dorsal patch of *L*. *yerbabuenae*.

**Fig 9:**
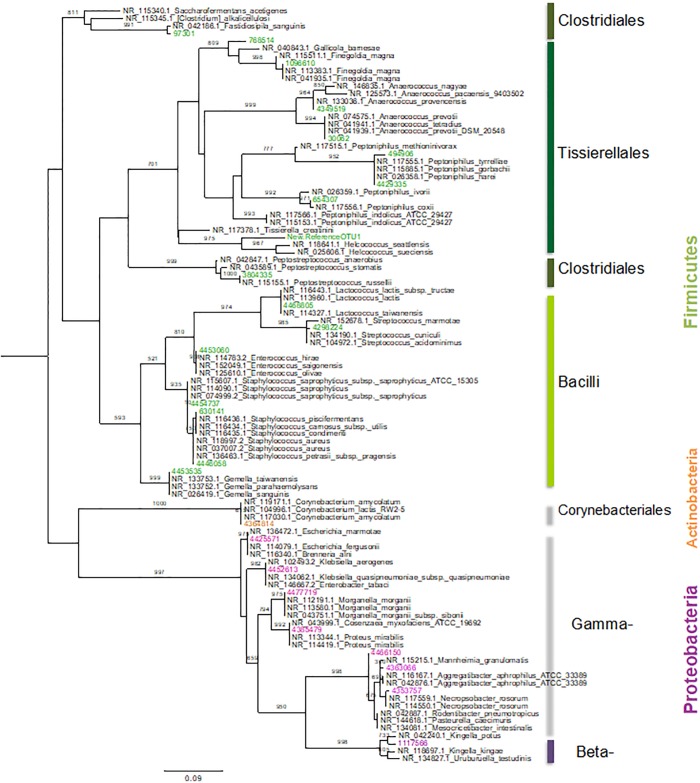
Taxonomic affiliation of each OTU to its three best references (RefSeq-NCBI Database). The code names of related species sequence, shows “NCBI accession id”_”species name”.

A subset of 26 OTUs was shared among all samples, and they collectively accounted for 30–75% of the microbial abundance of the dorsal patch per individual (Figs 7 and 8). Since these were all unassigned OTUs, we generated a phylogenetic tree was generated to associate these 26 shared bacteria to their closest relatives (RefSeq-NCBI Database) (Fig 9). These bacteria are distributed among 13 families; the four with the highest abundance were Tissierellaceae, Enterobacteriaceae, Pasteurellaceae and Streptococcaceae. Three Enterobacteriaceae genera were unassigned, along with one genus each from Pasteurellaceae, Nisseriaceae, Gemellaceae (see supporting information), and Planococcaceae. There was also one unassigned family.

The distribution pattern of 20 of these 26 bacteria was generally similar among samples. Only six showed differential abundance patterns: Pasteurellales OTUs 4466150 and 4353757, Clostridiales OTUs 97301, 768514, 3804335, 4349519 and Bacillales 4454737, 630141 and 4446058. OTUs for which we did not find a species-level relative were: *Clostridum* (97301), *Peptononiphilus* (494906; 4429335; 654307), *Anaerococcus* (4349519; 30062), *Gallicola* (768514), *Peptostreptococcus* (3804335), *Finegoldia* (1096610), *Helcococcus* (New.ReferenceOTU1), *Lactococcus* (4468805), Planococcaceae (630141), *Staphylococcus* (4446058), *Enterococcus* (4453060), Enterobacteriaceae (4425571, 4452613, 4477719, 4385479), Pasteurellaceae (4466150, 4363066, 4353757), Streptococcaceae (4298224), Neisseriaceae (1117566), Gemellaceae (4453535), unassigned (4454737), and Corynebateriaceae (4364814) (Figs 7 and 8).

## Discussion

### Dorsal patch core diversity

Although the dorsal patch of *L*. *yerbabuenae* males harbors a large microbial diversity, A subset of 26 OTUs was shared among all samples, and they collectively accounted for 30–75% of the microbial abundance of the dorsal patch per individual. Within this shared microbiome, 16 phylotypes are fermentative bacteria. These anaerobic bacteria are reported to be relevant in the production of short-chain fatty acids (SCFAs), which in several mammals have been shown to contribute to scent production that influences behavior, for example in bats (*Saccopteryx bilineata* [20] and *Leptonycteris yerbabuenae* [28]), hyenas (*Crocuta crocuta* and *Hyaena hyaena* [49]), and lions (*Panthera leo* [50]). Most of the shared bacteria from the dorsal patch belong to genera and families found and described in humans as part of the skin microbiome (*Finegoldia*, Pasteurellaceae), associated with wounds or infections (*Helcococcus*, *Enterococcus*) [49], or with the production of fermented products or volatile fatty acids (*Peptostreptococcus*, *Anaerococcus*, *Gallicola*, *Peptostreptococcus*, *Lactococcus*, Planococcaceae).

The shared OTUs are shown in the phylogenetic reconstruction, positioned nearest to their closest known relative (Fig 9). Two of the bacteria from the genus *Lactococcus* and *Streptococcus* are associated with wounds [49]. OTUs from the genera *Helcococcus*, *Aggregatibacter*, *Enterococcus*, and the family Corynebacteriaceae and one unassigned OTU include bacteria with pathogenic potential [50]. OTUs from the genus *Finegoldia* and one unassigned OTU were classified as double-function bacteria with fermentative functions [51]. One OTU was unassigned with unknown functions, and OTUs belonging to the genera *Gallicola*, *Anaerococcus*, *Peptoniphilus*, *Proteus*, unassigned *Staphylococcus*, *Clostridium*, *Peptostreptococcus* specialize in fatty acid production through fermentative processes [52]. More specific information on known functions of these groups is summarized in the supporting information (S1 Table).

### Diversity and potential implications of common bacteria in the dorsal patch of L. yerbabuenae

The difference observed in the Alpha and Beta diversity analyses of the bacteria (Table 1 and Fig 3) suggest individual-level variation in the assembly of the microbiota in the dorsal patch of each individual. Sixty-one percent of the shared OTUs in the dorsal patch have a fermentative function (Figs 7 and 8). These fermentative bacteria could metabolize short-chain fatty acids (SCFAs), likely contributing to scent production and possibly to mate attraction [17,53–55].

These results are consistent with the first premise of the fermentative hypothesis that volatile odorants are produced by bacteria that colonize mammal scent structures. This premise has also been supported by several other studies [17,20,54]. The second premise, that “individual “odor prints” result from the differences among individuals in the composition of the bacterial communities of these structures” was not tested in this study [20]. To do that, experimental studies are needed to detect the presence of SCFAs and confirm their role in individual recognition.

The recognition of skin microbiota as a main component of odor production is key to further understanding animal behavior. The olfactory receptors, the main olfactory epithelium, and the vomeronasal organ receive the odor signals produced by bacteria, which are then sent to the brain, a chemical signal pathway known as the microbiome-skin-brain axis [55]. The presence of fermentative bacteria in the dorsal patch of *L*.*yerbabuenae* could be important in generating odor, a potential reproductive signal.

## Conclusion

This study contributes to the establishment of baseline knowledge of the bacteria associated with the dorsal patch of *L*. *yerbabuenae* using molecular methods (16S rRNA sequencing). High-throughput sequencing techniques, along with bioinformatic analysis, allowed us to describe the biodiversity of the bacterial consortium of the dorsal patch of *L*. *yerbabuenae*. Current microbiome analyses are based on genetic diversity and composition estimations, coupled with information found in the literature. However, a major remaining challenge is that the large majority of the sequences found in this study were unassigned OTUs, making it necessary to rely on assertive association techniques using phylogenetic trees and other informatic comparisons to understand the system. This information could be substantially deepened by using a metagenomic approach to infer functional and taxonomic information as well as metabolic properties present in the bacterial communities associated with this and other systems, as well as their repercussions in the host and its life cycle. There is a long journey ahead to understand how microbiomes intervene in diverse biological systems, and how they contribute to regulating host behavior and reproduction through chemical signaling.

## Supporting information

S1 Table Functions associated with the 26 shared OTUs found in L. yerbabuenae dorsal patch. (ZIP)
