# Supplementary material for: Microbiota composition of the dorsal patch of reproductive male Leptonycteris yerbabuenae
Source: PLoS One. 2019 Dec 16;14(12):e0226239. doi: 10.1371/journal.pone.0226239 (PMC6913938; doi:10.1371/journal.pone.0226239)
Supplement: S1 Table — (ZIP) [file pone.0226239.s001.zip › S1Table.docx]

| **FAMILY** | **GENUS** | **OTU IDs** | **ENERGETIC METABOLISM** | **DESCRIPTION** | **Key References** |
| --- | --- | --- | --- | --- | --- |
| Tissierellaceae | *Gallicola* | 768514 | O-A | Acetic and lactic acids metabolized from Peptone/Yeast/Glucose | Ezaki et tal 2001 |
|  | *Anaerococcus* | 4349519  30062 | O-A | Metabolization of peptones and amino acids. The major end-products are butyric acid, lactic acid, propionic and succinic acids. Most species are able to ferment several carbohydrates, glucose, fructose, sucrose and lactose as main fermentative sugars.  . |  |
|  | *Peptoniphilus* | 654307  4429335  494906 | O-A | Butyrate-producing, use peptone and amino acids |  |
|  | *Helcococcus* | New. ReferenceOTU1 | F-A | Could be an avirulent member of the skin flora, like other microorganisms of low pathogenic potential. | Caliendo et al 1995 |
|  | *Finegoldia* | 4364814 | F-A | Produce acids using fructose and glucose. Part of the normal flora of the gastrointestinal and female urogenital tract and probably of the skin. Frequently isolated from human pathological specimens, particularly infections of skin, soft tissue, bone and joint. | Murdoch and Shah 1991 |
| Enterobacteriaceae | *Proteus* | 4385479 | F-A | Ferment glucose and produce acids – Reduce nitrates into nitrites | Mohr et al 2000 |
|  | Unassigned | 4477719 | F-A |  |  |
|  | Unassigned | 4452613 | F-A |  |  |
|  | Unassigned | 4425571 | F-A |  |  |
| Pasteurellaceae | Unassigned | 4353757 | F-A | Opportunistic, secondary invaders which under normal conditions coexist with the animal host on mucous membranes of the upper respiratory, alimentary and lower genital tracts. Able to cause infections under predisposing circumstances  , | Dousse et al 2008 |
|  | *Aggregatibacter* | 4363066  4466150 | F-A |  | Dousse et al 2008 |
| Streptococcaceae | *Lactococcus* | 4468805 | F-A | Produce lactic acid and aromatic compounds including alcohols, ketones, and aldehydes. Some produce antimicrobial compounds (bacteriocin, nisin and lactococcin, polyphenolic compounds). | Smit et al 2005 |
|  | *Streptococcus* | 4298224 | F-A |  |  |
| Neisseriaceae | Unassigned | 1117566 | S-A | Can break through the mucosal barriers to cause septicemia. Contribute to natural protection against *N. meningitidis* infection, helping to develop natural immunity. Lactose fermentation | Hollis et al 1970 |
| Enterococcaceae | *Enterococcus* | 4453060 | F-A | Cause urinary, wound, intraabdominal, and pelvic infections, superinfections and bacteremia (often together with other organisms) | Murray 1990 |
| Gemellaceae | Unassigned | 4453535 | O-A | Found in oral cancer. | Guerrero-Presto et al 2016 |
| Planococcaceae | Unassigned | 630141 | A to F-A | Iso-methyltetradecanoic acid, anteiso-methyltetradecanoic acid is the major fatty acids present in all genera. | Shivaji et al 2014 |
| Staphylococcaceae | *Staphylococcus* | 4446058 | A | Produce teichoic acids using glucose. Commonly found in mammal skin tissue. | Schleifer and Kloos 1975, Marsilio et al 2018 |
| Clostridiaceae | *Clostridium* | 97301 | O-A | Cellulose and hemicellulose biomass degradation and carbon fixation. Fermentative subsets of these taxa are selectively stimulated by glucose and might therefore be capable of consuming mucus and plant-derived saccharides during gut passage. | Wüst et al 2011 |
| Peptostreptococcaceae | *Peptostreptococcus* | 3804335 | A | Carbohydrate fermentation reactions and production of saccharolytic and proteolytic enzymes. Member of the normal oral and vaginal flora. | Murdoch 1998 |
| Actinomycetales | Corynebacteriaceae | 4364814 | F-A | Opportunistically capitalize on atypical access to tissues (via wounds) or weakened host defenses | Yeager et al 2017 |
| Unassigned | Unassigned | 4454737 | - |  |  |

Gram-positive (G-P), Gram-negative (G-N), Gram-variable (G-V), Obligate Anaerobic (O-A), Facultative Anaerobic (F-A), Strictly Anaerobic (S-A), Anaerobic (A)

S1 Table. Functions associated with the 26 shared OTUs found in *L. yerbabuenae* dorsal patch.
